# The PCOC Symptom Assessment Scale (SAS): A valid measure for daily use at point of care and in palliative care programs

**DOI:** 10.1371/journal.pone.0247250

**Published:** 2021-03-25

**Authors:** Barbara A. Daveson, Samuel Frederic Allingham, Sabina Clapham, Claire E. Johnson, David C. Currow, Patsy Yates, Kathy Eagar

**Affiliations:** 1 Palliative Care Outcomes Collaboration, Australian Health Services Research Institute, University of Wollongong, Wollongong, New South Wales, Australia; 2 Monash Nursing and Midwifery, Monash University, Eastern Health, Victoria, Australia; 3 Faculty of Health, University of Technology Sydney, Ultimo, New South Wales, Australia; 4 Centre for Palliative Care Research and Education, Queensland Health, School of Nursing, Queensland University of Technology, Kelvin Grove, Queensland, Australia; University of Bristol, UNITED KINGDOM

## Abstract

**Background:**

Very few measures are used successfully as part of routine care within national palliative care outcome programs. Only a handful of studies examine these measures. The aim of this study is to evaluate the validity of a measure used in a national outcomes program: the Palliative Care Outcomes Collaboration Symptom Assessment Scale (PCOC SAS).

**Methods:**

A retrospective multi-site cohort study with secondary analysis of routinely collected patient-level data to assess PCOC SAS’s internal consistency, construct validity, reliability, interpretability, acceptability and sensitivity. The analyses used two sets, with data collected by inpatient and community palliative care services registered with the Australian national PCOC.

**Results:**

Dataset one included 1,117 patients receiving palliative care from 21 services. Dataset two included 5,294 patients receiving palliative care from 119 PCOC services. PCOC SAS demonstrated the ability to detect and discriminate distress by palliative care phase, functional status and diagnosis. Excellent and good convergent and discriminant validity were demonstrated. Fair through to substantial inter-rater and intra-rater reliability levels were evidenced. Sufficient interpretability resulted along with necessary levels of acceptability and sensitivity.

**Conclusion:**

PCOC SAS is a valid and reliable patient-reported outcome measure suitable for use in routine clinical care with patients requiring palliative and or end-of-life care, including in national outcomes programs.

## Introduction

Estimates of the global burden of health-related suffering show that the number of people that may benefit from palliative care will almost double by 2060. This burden, driven by rises in cancer deaths and followed by increases in cerebrovascular disease and lung disease, will increase most rapidly in older adults (≥70 years) [1]. This changing and challenging demography will place increasing demands on services and health systems around the world.

Understanding local and population need is essential to responding to this global burden, and patient-reported outcome measures (PROMs) can help with this. At a local level, PROMs immediately help healthcare professionals and clinical teams identify which outcomes to prioritise, while national PROM programs can help develop a broader population perspective useful to health systems planning [2]. Combined, local and national perspectives help inform a coherent response to this growing burden.

In Australia, an integrated national palliative care outcomes program, the Palliative Care Outcomes Collaboration (PCOC), has shown to be feasible, desirable and useful in systematically improving outcomes in patients receiving palliative care. This has been demonstrated at a local, sub-national and national level [3]. The PCOC initiative has shown that nationally agreed-upon measures can identify unwarranted variations in outcomes and support systems-level improvements. Assessing the outcomes of patients as part of routine care is integral to the PCOC national program, with the program being adopted in other national settings, including countries within the European and the Western Pacific regions.

A measure central to PCOC is the Symptom Assessment Scale (SAS). The version of SAS first used by PCOC was developed by Linda Kristjanson and others in the 1980s and 1990s [4]. Since then, SAS has been shown to have moderate internal consistency, good-to-excellent test-retest reliability, and sensitivity to change in the following items: appetite, insomnia, nausea and pain in people with advanced cancer [5]. SAS has also been shown to be a feasible measure as part of routine palliative care [3]. It is used nationally in Australia, and locally and or nationally in a range of countries including in Ireland, Germany, New Zealand, Taiwan, Japan and Singapore. The measure and its accompanying supporting documentation has been translated into 15 languages.

SAS has recently been developed to further enhance its properties for use at point of care. The aim of this study was to examine this new version of the SAS instrument, referred to as the PCOC SAS. In particular, this study aimed to examine PCOC SAS’s psychometric properties in a large representative population, building upon existing knowledge of the earlier version of the instrument.

## Methods and materials

### Study design

A retrospective, multi-site, cohort study involving secondary analysis of routinely collected was completed to test the psychometric properties of PCOC SAS. Well-established criteria for validating measures was used [6], drawing upon previously and recently published approaches within the context of palliative and end-of-life care [7, 8]. This study therefore involved the evaluation of: internal consistency, construct validity, reliability (agreement, inter-rater and intra-rater reliability), interpretability, sensitivity and acceptability (Table 1).

**Table 1:** A brief explanation of the key psychometric properties examined in this study.

Psychometric property	Brief explanation
Internal consistency	Internal consistency examines how items (e.g., questions) in a measure (e.g., PCOC SAS) are associated (or correlate) with each other. Internal consistency provides an indication of the level of coherence of a measure. It also provides an indication of whether there are redundant items in a measure. From a clinical perspective, good internal consistency for measures that examine a single concept is important. This is because measures with high internal consistency may place unnecessary burden on patients, carers and staff (as the measure may have redundant questions in it). While measures with a low internal consistency may mean that a number of different concepts are being measured by the scale, and this may make it more challenging to use as part of routine clinical care.
Construct validity	Construct validity investigates whether a measure examines the concept (or construct) it intends to. Convergent and discriminant validity are part of construct validity. Convergent validity tests how closely items correlate. Discriminant (or divergent) validity recognises that unrelated items should have low correlations. Theory or hypothesis testing is part of examining these types of validity. This is because what we need to measure may not always be observed directly. Establishing a clear hypothesis or theory to test from the beginning is important as it helps reduce the risk of bias. If the relationship between the theory and the measure is not apparent in the results from the study, then the measure does not measure what it intends to measure (and it has poor construct validity) or the theory tested was incorrect. Reporting negative findings and reviewing the theories that were tested in research is an important part of developing outcome measures. This helps build knowledge, allows for critical appraisal of research results, and it ensures honesty in research reporting and conduct.
Reliability	Concerns whether a measure is able to produce reproducible and consistent results. Intra-rater (test-retest) reliability involves the same person repeating the measure. Inter-rater reliability examines agreement between different raters (e.g., a carer and a staff member).
Interpretability	Provides an indication of the extent to which someone (the patient, the carer, staff) can derive meaning from the numerical scores in the measure. This is important when it comes to ensuring the measure informs clinical care.
Acceptability	Examines how agreeable the measure is to the user (the patient, the carer, staff). Low levels of acceptability may result in a measure not being used, it being used incorrectly and missing data.
Sensitivity	Examines whether the measure can detect differences between groups. For example, whether the measure can detect differences between different diagnostic groups or those with advanced versus early-stage disease.

Acknowledgment: This information has been derived from: Terwee CB, Bot SD, de Boer MR, van der Windt DA, Knol DL, Dekker J, et al. Quality criteria were proposed for measurement properties of health status questionnaires. Journal of clinical epidemiology. 2007; 60(1):34–42 and Fayers PM, Machin D. Scores and measurements: validity, reliability, sensitivity. Quality of life: the assessment, analysis and interpretation of patient reported outcomes. 2nd ed. Chichester: John Wiley & Sons; 2007. pp. 77–108.

### Materials

PCOC SAS is a patient-reported scale used to measure subjective aspects related to health. It evaluates perceived distress. Although designed to be used by patients from any disease or treatment group and age, it can be used by proxies [9]. The scale assesses eight dimensions: pain, insomnia (difficulty sleeping), nausea, bowel problems, appetite problems, breathing problems, fatigue, and an ‘other’ item, which may be added to the measure.

For each of the eight dimensions, there are 11 levels in the response options. The response options range from ‘absent’ to ‘severe’ distress. A score of 0 indicates that the patient is distress free, meaning that the score >0 effectively identifies distress for each domain in the scale. In order to assist patients to discriminate between these 11 response options reliably, the PCOC SAS response options have been grouped into six intensity categories. Each category has a corresponding descriptor, colour and facial expression. Higher total scores and higher individual item scores represent higher levels of distress [S1 File].

In relation to the use of PCOC SAS as part of routine care, all Australian PCOC-registered services use the measure daily within inpatient settings (including daily in residential aged care facilities) and at each contact within community settings. Its use is bolstered by a national education program, online educational materials, and calibration sessions, supported by a clinical manual and information brochure [10]. PCOC SAS and its accompanying information brochure is available in 15 languages: Arabic, Chinese (simplified and traditional), Croatian, English, Filipino, Germany, Greek, Hindi, Italian, Macedonian, Russian, Serbian, Somali, Spanish and Vietnamese. Throughout Australia, PCOC SAS scores are either collected using paper or electronic means (e.g., portable handheld devices). PCOC provides IT software to participating services to help address any technological barriers to its use at point of care.

### Population and settings

All services registered with PCOC were deemed eligible to participate in this study. A smaller set of services were approached to participate in a sub-study to allow for a more thorough examination of the measure. These sub-study services were services able to adequately represent national palliative care services and the population currently accessing palliative care (including public and private providers). A further criterion was that services with well-established procedures for recording who completed symptom distress assessments could participate. Hospital-based services that only offered consult-liaison services were excluded due to the advisory nature of the service.

### Data collection

Demographic information collected for patients at baseline included: age, sex, country of birth, preferred language, Indigenous status, and primary diagnosis. Clinical patient information collected included: primary diagnosis, palliative care phase [11, 12], Australia-modified Karnofsky Performance Status (AKPS) [13], the Palliative Care Problem Severity Score (PCPSS) [14], the Resource Utilisation Group–Activities of Daily Living (RUG-ADL) [12] and PCOC SAS [5]. Data was collected for a four-week period commencing October 2017.

### Ethics

Ethical approval was granted for the PCOC program by the University of Wollongong and Illawarra Shoalhaven Local Health District Health and Medical Human Research Ethics Committee (reference no. HE2006/045). This involved an amendment to the original PCOC ethics application to ensure approval of the analysis of patient and proxy data, which was not routinely submitted to PCOC although routinely documented by some services. As only routinely collected, de-identified, aggregated clinical data were used in this study, separate participant consent was not necessary.

### Analysis

Secondary analysis of patient-level data collected as part of routine clinical practice was completed. Descriptive statistics were derived to describe the patient sample, the range of individual item PCOC SAS scores, the PCOC SAS total score, and missing values.

#### Internal consistency

We estimated the internal consistency of PCOC SAS using Cronbach’s α for the total score and individual items. Due to the multi-dimensional nature of the measure, and before analysis, we lowered the normally accepted threshold for good internal consistency from 0.8 to 0.6 [15].

#### Construct validity

For construct validity, we examined PCOC SAS against phase and functional status. We hypothesised that patients in an ‘unstable’ or ‘deteriorating’ phase would have higher PCOC SAS total scores, as compared to those in ‘stable’ and ‘terminal’ phase. In line with established definitions, a stable palliative care phase was defined as the patient’s symptoms and problems were adequately controlled by established management. An unstable palliative care phase was when an urgent change in the patient’s plan of care or their emergency treatment was required due to a new problem or an escalation of an existing patient or carer problem. A patient was defined as being in a deteriorating palliative care phase when their care plan was adequate but periodic review was required due to gradual functional decline and or a worsening of existing problems, and or the development of new but expected problems (either patient or carer problems). A terminal palliative care phase was when the death of the patient was likely in a matter of days [11, 14]. Also, we hypothesised that patients with a lower AKPS [13] who would have lower PCOC SAS scores as compared to those with higher AKPS scores.

For convergent and discriminant validity (part of construct validity), we used Spearman’s correlation coefficient to correlate PCPSS with PCOC SAS. We hypothesised that a high correlation (r>0.70) would be evident for the pain items on both measures. Given the differences between the measures (PCOC SAS examines distress and the PCPSS examines symptom severity), we anticipated mid-range correlations between the total scores. Mid-range correlations were anticipated between the PCPSS ‘other’ item and the PCOC SAS total score, excluding the pain item. Mid-range correlations were defined in advance of analysis as r = >0.5⩽0.7.

#### Reliability

For inter-rater reliability, we analysed independent patient and proxy ratings completed ≤48 hours when the patient was in the stable phase. For patients in an unstable or deteriorating phase, this was analysed between patient and proxy ratings completed ≤24 hours of each other. Cohen’s weighted kappa was calculated for reliability. For agreement, both exact agreement and a tolerance agreement, reflecting the proportion of cases where proxy or patients’ ratings were equal to or within +1 or −1 of the score, were calculated. Cohen’s Weighted Kappa was calculated to ascertain the level of chance-corrected agreement. A pre-established criterion of agreement was used: poor k<0.00, slight k = 0.00−0.20, fair k = 0.21−0.40, moderate k = 0.41−0.60, substantial k = 0.61−0.80, almost-perfect k = 0.81−1.00 [16].

We hypothesised that test–retest reliability (intra-reliability) would be demonstrated in a patient with no change in their phase within one episode of care, with analysis by phase undertaken. Pearson’s correlation coefficient was used to examine incidences where PCOC SAS was completed twice ≤48 hours when the patient was in a stable phase. Analysis of the instances where assessments were completed ≤24 hours for when the patient was in an unstable or deteriorating phases. Patients in terminal phases were excluded (due to the likelihood of a larger proportion of proxy reported assessments).

#### Interpretability

A Flesch-Kincaid readability test [17] was completed to identify the years of education required to complete PCOC SAS. In line with pre-established standards, sufficient interpretability was evident if someone with the reading skills of a ≤12-year old were required [18]. Pre-established standards to define what was acceptable for the extent of missing items were used, with items with values of >4% deemed to be insufficient [15].

#### Acceptability

Acceptability was examined by analysing the distribution of the PCOC SAS total scores for each patient (median, interquartile range (IQR)). Acceptability was hypothesised to be sufficient if PCOC SAS total scores were well distributed and median scores were near the mid-point of the scale. Floor or ceiling effects would be evident if more than 15% of respondents achieved the lowest or highest possible score, respectively [6]. Acceptability was examined by phase, individual items and total scores. (The levels of missing data, which may also be an indication of acceptability, is reported in the interpretability section.)

#### Sensitivity

In relation to sensitivity, we hypothesised that PCOC SAS would discriminate between individual symptom distress scores by diagnosis and phase. We anticipated that as compared to all other patients diagnosed with cancer, higher distress scores would be found for patients diagnosed with: lung cancer or COPD for the breathing item, colorectal cancer for the bowel item, head and neck cancer for pain, pancreatic cancer for pain, gynaecological cancer for pain, bone and soft tissue cancers for pain, and that there would be more reports of distress related to fatigue in deteriorating and unstable phases.

## Results

### Palliative care service and subject characteristics

Two sets of data were derived for this analysis. Dataset one included 1,117 patients receiving palliative care from 21 services. Of the 21 services, 12 were inpatient services and nine provided services in the community. Of the 1,117 patients, the majority (n = 819, 72.4%) were receiving care in the community, with just over a quarter (n = 320, 28.6%) receiving care in an inpatient setting. Dataset two included 5,294 patients receiving palliative care from 119 services. Of the 119 services, 39 services were providing palliative care in community and 80 inpatient services. In a few instances, patients received care in both the inpatient and hospital setting and so are counted against both, meaning that percentages will not sum to 100% (Table 2). Across both datasets, patients with a primary diagnosis of cancer formed the majority. Approximately 1 in every 5 patients had a non-malignant primary diagnosis with respiratory failure being the most common, followed by those diagnosed with cardiovascular disease.

**Table 2:** Characteristics of the patients involved in the study (N = 1,117) compared to the entire PCOC cohort for the comparable time period (N = 5,294).

	Study 1 cohort n (%) (N = 1,117)	Study 2 PCOC cohort n (%) (N = 5,294)
**Sex**		
Male	588 (52.6)	2,742 (51.8)
Female	523 (46.8)	2,552 (48.2)
Missing	6 (0.5)	0 (0.0)
**Country of birth**		
Australia	610 (54.6)	3,213 (60.7)
Other	484 (43.3)	1,968 (37.2)
Missing	23 (2.1)	113 (2.1)
**Preferred language**		
English	969 (86.8)	4,752 (89.8)
Other	122 (10.9)	501 (9.5)
Missing	26 (2.3)	41 (0.8)
**Indigenous status**		
Aboriginal and/or Torres Strait Islander	16 (1.4)	79 (1.5)
Neither Aboriginal nor Torres Strait Islander	1,072 (96.0)	5,097 (96.3)
Missing	29 (2.6)	118 (2.2)
**Life-limiting illness**		
Cancer diagnosis	846 (75.5)	4128 (78.0)
Non-cancer diagnosis	262 (23.5)	1,158 (21.9)
Missing	9 (0.8)	8 (0.2)
**Age group (years)**		
0–34	17 (1.5)	56 (1.1)
35–44	34 (3.0)	116 (2.2)
45–54	86 (7.7)	362 (6.8)
55–64	172 (15.4)	869 (16.4)
65–74	278 (24.9)	1,364 (25.8)
75–84	293 (26.2)	1,429 (27.0)
85–94	202 (18.1)	961 (18.2)
95+	29 (2.6)	110 (2.1)
Missing	6 (0.5)	27 (0.5)

The full range of individual item PCOC SAS scores and the PCOC SAS total scores were evident in the full and smaller datasets. The patient-reported values for the smaller dataset are conveyed in Table 3. Limited missing values were found for all items (i.e., ≤4%), except for insomnia (Table 3).

**Table 3:** Descriptive statistics and distribution of PCOC SAS total scores and individual items, including by distress status.

	Total scores	Pain	Fatigue	Breathing	Bowels	Nausea	Appetite	Insomnia
**Evaluable PCOC SAS** (n = 9,821)								
PCOC SAS recorded, n (%)	7,185 (73.2)	9,746 (99.2)	9,663 (98.4)	9,643 (98.2)	9,633 (98.1)	9,672 (98.5)	9,627 (98.0)	7,236 (73.7)
PCOC SAS with one or more items missing, n (%)	2,636 (26.8)	75 (0.8)	158 (1.6)	178 (1.8)	188 (1.9)	149 (1.5)	194 (2.0)	2,585 (26.3)
**Absent distress**								
Distress absent n (%)	646 (9.0)	3,852 (39.5)	3,188 (33.0)	5,727 (59.4)	7,192 (74.7)	7,915 (81.8)	6,211 (64.5)	5,463 (75.5)
Distress present n (%)	6,539 (91.0)	5,894 (60.5)	6,475 (67.0)	3,916 (40.6)	2,441 (25.3)	1,757 (18.2)	3,416 (35.5)	1,773 (24.5)
**Distress present–PCOC SAS summary statistics**								
Median (IQR)	7 (4−11)	2 (1−4)	3 (2−4)	2 (2−4)	2 (1−3)	2 (1−3)	2 (1−3)	2 (1−3)
Range	1–10	1–10	1–10	1–10	1–10	1–10	1–10	1−10
**Distress present–PCOC SAS distribution % (n)**								
Total score	(n = 6,539)	(n = 5,894)	(n = 6,475)	(n = 3,916)	(n = 2,441)	(n = 1,757)	(n = 3,416)	(n = 1,773)
**1**	3.3	25.3	12.3	22.7	31.0	33.8	25.2	26.4
**2**	8.0	28.1	25.4	30.7	29.7	26.0	34.6	32.7
**3**	7.8	18.8	22.1	20.2	16.6	17.0	19.3	18.8
**4**	9.1	10.5	15.3	10.8	9.1	8.7	8.8	9.8
**5**	8.0	7.1	11.8	7.4	7.1	7.9	5.4	6.8
**6**	9.1	4.1	6.7	3.9	3.1	3.2	2.8	2.7
**7**	7.2	2.4	3.9	2.3	1.3	1.5	1.5	1.1
**8**	7.0	2.3	1.8	1.4	1.3	1.5	1.7	1.4
**9**	5.8	0.8	0.5	0.4	0.5	0.3	0.3	0.2
**10**	6.5	0.6	0.2	0.2	0.3	0.1	0.3	0.1
**11–59**	27.9	N/A	N/A	N/A	N/A	N/A	N/A	N/A
**60–70**	0.0	N/A	N/A	N/A	N/A	N/A	N/A	N/A

### Internal consistency

With rounding conventions applied, the PCOC SAS demonstrated good internal consistency with a Cronbach α of 0.59 for patient ratings, and 0.62 when all assessments evaluated, that is with patient and proxy-rated assessments examined together.

### Construct validity

Our analysis confirmed our *a priori* hypothesis regarding PCOC SAS and phase, with higher PCOC SAS total scores observed for unstable and deteriorating phases, as compared to total scores for stable and terminal phases. This was also observed for each individual PCOC SAS item. Higher percentages of assessments revealed more instances of patients being distress free when in stable (21.5%) and terminal (54.4%) phases, as compared to unstable (13.8%) and deteriorating (14.4%) phases.

Our hypothesis that those with a lower AKPS would have lower PCOC SAS scores was not observed as those with higher and lower AKPS scores reported less distress. A U-curve for the percentage of assessments was found for the PCOC SAS item scores (excluding the ‘other’ item) when compared with the AKPS item scores (Table 4).

**Table 4:** PCOC SAS patient-rated assessments: Median total score distress-free total scores (%), and distress-free item scores by palliative care phase and functional status (AKPS).

	Total PCOC SAS scores		Percentage of distress-free PCOC SAS item scores						
	Median	Percentage distress-free	Pain	Fatigue	Breathing	Bowels	Nausea	Appetite	Insomnia
**Palliative care phase**									
Stable	5	12.9	48.9	39.9	63.2	79.9	87.8	72.6	80.5
Unstable	11	5.1	23.4	29.1	58.4	68.5	62.8	53.1	67.7
Deteriorating	9	2.9	27.4	21.6	53.8	68.7	75.0	52.5	68.7
Terminal	5	22.8	41.4	60.8	61.6	78.2	83.7	88.4	81.0
**AKPS**									
100	0	66.7	83.3	83.3	100.0	100.0	100.0	100.00	100.0
90	2	16.3	65.5	55.3	84.7	83.5	92.9	82.4	90.0
80	4	13.1	45.0	35.6	75.1	80.1	87.2	66.8	78.3
70	6	8.2	40.4	33.2	67.5	78.1	83.9	63.8	78.1
60	7	6.6	41.8	30.1	55.3	76.5	82.7	62.1	71.6
50	7	8.0	38.6	33.6	51.4	72.0	80.5	62.1	70.8
40	7	11.3	35.3	29.5	60.6	74.3	79.7	64.0	76.1
30	8	10.4	33.4	32.5	59.8	66.8	77.4	64.0	78.5
20	4	10.6	43.9	42.5	72.7	75.1	86.4	81.6	90.0
10	2	36.6	44.4	74.6	81.0	79.3	91.4	94.6	95.5

Calculation of Spearman’s rank-sum correlation coefficient to examine correlations between the PCPSS with PCOC SAS scores indicated a high correlation between the pain items on both measures (r = 0.76, p = < 0.001). As anticipated, mid-range correlations between the PCOC SAS total score (excluding pain) and the PCPSS ‘other symptoms’ score (r = 0.55, p = < 0.001), and the total scores of PCOC SAS and the PCPSS were found (r = 0.59, p = < 0.001), with rounding conventions applied.

#### Reliability

Moderate and substantial levels of inter-rater reliability were observed for all PCOC SAS items (Table 5). The exception was the bowel item in the stable phase, and insomnia in the unstable and deteriorating phases. However, the percentage of exact (76.8%) and tolerance agreements (85%) for the bowel item was high in the stable phase, indicating substantial reproducibility. Similarly, the level of agreement was very good for the insomnia item, with an acceptable exact (71.3%) and tolerance (75.9%) agreements. Of note, the 95% CI for the insomnia item was relatively larger than all other items (95% CI 0.08–0.48). The sample size for this item was 100 patients (Table 5). In relation to test-retest (intra-rater) reliability, moderate to substantial agreement on all items resulted. The range of the 95% CI for the insomnia item remained similar in breadth to the inter-rater reliability, however there was a larger number of exact and tolerance agreements (Table 6).

**Table 5:** Inter-rater reliability of PCOC SAS: Types and levels of agreement between patient and proxy ratings by phase and PCOC SAS items.

Palliative care phase	PCOC SAS item	N	Exact agreement %	Tolerance Agreement^+^ %	Weighted kappa	95% CI	Level of agreement
**Stable, unstable and deteriorating**	Pain	544	52.4	70.2	0.55	0.39–0.52	Moderate
	Fatigue	537	67.0	77.7	0.5	0.46–0.60	Moderate
	Breathing	536	79.9	89.6	0.6	0.56–0.71	Substantial
	Bowel problems	533	73.4	84.2	0.4	0.32–0.49	Moderate
	Nausea	535	86.7	91.4	0.5	0.39–0.61	Moderate
	Appetite problems	534	85.6	89.0	0.5	0.33–0.56	Moderate
	Insomnia	390	83.3	88.2	0.4	0.26–0.53	Moderate
**Stable**	Pain	370	55.7	73.2	0.4	0.35–0.51	Moderate
	Fatigue	368	69.0	79.6	0.5	0.38–0.56	Moderate
	Breathing	368	82.1	91.8	0.6	0.53–0.73	Substantial
	Bowel problems	367	76.8	85.0	0.3	0.22–0.44	Fair
	Nausea	367	91.0	94.6	0.4	0.23–0.58	Moderate
	Appetite problems	366	89.9	92.9	0.5	0.30–0.62	Moderate
	Insomnia	282	87.9	92.9	0.5	0.30–0.64	Moderate
**Unstable and deteriorating**	Pain	174	45.4	63.8	0.4	0.33–0.53	Moderate
	Fatigue	169	62.7	73.4	0.6	0.46–0.67	Moderate
	Breathing	168	75.0	84.5	0.6	0.52–0.75	Moderate
	Bowel problems	166	65.7	82.5	0.5	0.36–0.62	Moderate
	Nausea	168	77.4	84.5	0.5	0.40–0.67	Moderate
	Appetite problems	168	76.2	80.4	0.4	0.24–0.57	Moderate
	Insomnia	100	71.3	75.9	0.3	0.08–0.48	Fair

^+^ Tolerance agreement reflects the proportion of cases where proxy and patient ratings were equal to or within +1 or −1 of each other.

**Table 6:** Intra-rater reliability of PCOC SAS: Types and levels of agreement in repeat patient-reported assessments.

Palliative care phase	PCOC SAS item	N	Exact agreement %	Tolerance agreement %	Weighted kappa	95% CI	Level of agreement
**Stable, Unstable and deteriorating**	Pain	1,135	63.3	78.0	0.6	0.54–0.63	Moderate
	Fatigue	1,123	64.6	78.5	0.6	0.57–0.65	Moderate
	Breathing	1,121	84.7	90.9	0.7	0.65–0.75	Substantial
	Bowel problems	1,121	80.5	87.2	0.6	0.50–0.62	Moderate
	Nausea	1,128	86.4	91.2	0.6	0.49–0.63	Moderate
	Appetite problems	1,121	86.6	93.0	0.7	0.63–0.74	Substantial
	Insomnia	1,067	86.6	90.5	0.6	0.53–0.67	Substantial
**Stable**	Pain	1,010	66.0	80.5	0.6	0.52–0.62	Moderate
	Fatigue	1,005	65.6	78.7	0.6	0.54–0.64	Substantial
	Breathing	1,004	85.5	91.4	0.7	0.64–0.75	Substantial
	Bowel problems	1,005	82.0	88.6	0.6	0.52–0.65	Moderate
	Nausea	1,008	88.2	92.8	0.6	0.48–0.65	Moderate
	Appetite problems	1,004	88.8	94.9	0.7	0.66–0.78	Substantial
	Insomnia	967	87.7	91.5	0.6	0.54–0.69	Substantial
**Unstable and deteriorating**	Pain	125	41.6	57.6	0.5	0.38–0.61	Moderate
	Fatigue	118	55.9	77.1	0.6	0.54–0.75	Substantial
	Breathing	117	76.1	86.3	0.7	0.62–0.82	Substantial
	Bowel problems	116	67.2	75.9	0.4	0.27–0.58	Moderate
	Nausea	120	71.7	78.3	0.5	0.35–0.65	Moderate
	Appetite problems	117	67.5	76.9	0.5	0.35–0.64	Moderate
	Insomnia	100	76.0	81.0	0.5	0.33–0.73	Moderate

1 Tolerance agreement reflects the proportion of cases where a subsequent patient ratings was the same as or within +1 or −1 of an immediately following score.

#### Interpretability

Testing of the measure showed sufficient interpretability. The Flesch reading ease was 86.2 (grade six level USA education i.e., 11−12 years old). The Flesch-Kincaid grade level was 4.6. Acceptable levels of missing data (i.e., ≤4%) was found on all items, except the insomnia item.

#### Acceptability

After removal of the screening response option of 0, evaluation of the distribution of the PCOC SAS scores indicated that the values were near the midpoint of the scale, especially for pain, fatigue and breathing. The distribution was skewed. No floor or ceiling effects were found.

#### Sensitivity

We found that that PCOC SAS could discriminate between individual symptom distress scores by phase. In relation to diagnosis, we observed that patients diagnosed with lung cancer or COPD had higher scores of distress for breathing as compared to all other patients diagnosed with cancer. Patients with GIT cancers were found to report higher levels of distress for the nausea item, when compared to all patients diagnosed with cancer. No other statistically significant differences were found for the disease group comparisons (Table 7). There were more reports of distress related to fatigue in patients in unstable and deteriorating phases (Table 4).

**Table 7:** Comparisons between selected diagnostic groups: Kruskal-Wallis test results.

Diagnostic group of interest (n)	Comparison group (n)	Item of interest	Kruskal-Wallis Chi Square	Degrees of freedom	p-value
Lung cancer (n = 184)	All other cancers (n = 659)	Breathing	31.2	1	<0.001
Respiratory failure (n = 64)	All other non-malignant diagnoses (n = 197)	Breathing	35.0	1	<0.001
Colorectal cancers (n = 99)	All other cancers (n = 744)	Bowel	1.5	1	0.219
Colorectal cancers (n = 99)	All other cancers (n = 746)	Nausea	0.2	1	0.621
Other GIT cancers (n = 79)	All other cancers (n = 764)	Bowel	0.9	1	0.348
Other GIT cancers (n = 79)	All other cancers (n = 766)	Nausea	6.3	1	0.012
Head and neck cancers (n = 32)	All other cancers (n = 812)	Pain	0.1	1	0.790
Pancreatic cancers (n = 69)	All other cancers (n = 775)	Pain	0.0 (0.04)	1	0.847
Gynaecological cancers (n = 35)	All other cancers (n = 809)	Pain	2.4	1	0.123
Bone and soft tissue cancers (n = 11)	All other cancers (n = 833)	Pain	2.4	1	0.120

## Discussion

Our analysis confirms that PCOC SAS is a valid and reliable patient-reported outcome measure with sufficient interpretability for use as part of routine clinical care. It is sensitive enough to detect clinically relevant changes in patients with lung cancer, COPD and GIT cancers. It can be understood and comprehended by a 12-year old. Importantly, our findings were derived from information where PCOC SAS was used as part of routine clinical care with patients receiving palliative care. This applied health services research approach serves to strengthen the conclusions reported in our paper, which highlight the strong utility, feasibility and psychometric properties of PCOC SAS. Internationally, although several measures with good psychometric properties are being used by palliative care services within countries, very few measures that measure subjective states have demonstrated application in national palliative care outcome programs, with PCOC SAS being an exception.

One of the findings in our study that warrants further discussion is the levels of agreement between instances of patient reports. Moderate to substantial levels of agreement were observed for all PCOC SAS items. The exceptions were the bowel item in the stable phase, and insomnia in unstable and deteriorating phases. That acknowledged, the percentage of exact and tolerance agreements for these items were high, especially when considering the relatively large number of response options in PCOC SAS (11 response options). As the 95% CI for the insomnia item was larger than all other items and the sample size for this smaller, the item should be examined again with a larger sample size.

Missing data levels for PCOC SAS items were low, ranging from 0.8% for pain through to 2.0% for appetite. These levels are well below the level ≤4% usually deemed acceptable (i.e., ≤4%) [15]. They compare favourably to other measures in palliative care that assess subjective states [7]. The only exception to this was the insomnia item where very large volumes of data were missing (26.3%). Further investigation with services revealed that these missing values were due to an IT software usage issue, rather than a reflection of this item being missed as part of routine care or being of lower quality. Unlike other validation studies, our study was reliant on information collected as part of routine care, meaning these findings highlight the excellent extent of missing data for the PCOC SAS in routine care.

An additional novel finding was that national outcome measurement programs can be used to help develop measures for application with patients with advanced disease. We demonstrate that the testing of test-retest reliability is feasible when the concept of episode of care along with palliative care phase are combined to identify a period of stability in the patient. This is a useful finding as previously it has been suggested that the use of a 24- or 48-hour period of re-assessment for test-retest reliability may not be possible as palliative patients may change too much in this time period [7]. Our study shows that examining test-retest in routine practice is possible.

An unanticipated finding was the inverse relationship between performance status and distress in patients with advanced disease. We anticipated that those with lower AKPS scores would have higher reports of distress, as compared to those with higher AKPS scores. However, we found that reports of distress in patients with advanced disease are lower when they are able to function without impairment or with a great deal of impairment. This finding, whilst different to our hypothesis, is plausible, and many different theories may account for this unexpected finding. For example, a response shift may have occurred in these patients. That is, the patients may have adjusted their internal standard by which they measure their own distress. They may have changed the way they valued what was being measured or they may have even redefined their concept of distress as their illness continued to advance [19, 20]. An alternative explanation is that our finding may reflect the positive impact that services may have on helping patients achieving a response shift or symptom relief [21].

A major strength of our study is the large volume of evaluable data retrieved from routine clinical care involving 80% of specialist palliative care providers across a country, which arguably helps reduce selection bias. This means, for example, that patients with cognitive challenges were still included in the study, rather than deemed ineligible in a research study, for example. However, at the same time, the unexpected finding regarding the inverse relationship between performance status and distress, and the missing data in relation to the insomnia item are illustrative of the limitations inherent with this approach. Nevertheless, even despite skewness in distributions of the SAS total score and individual items, the statistical tests applied have resulted in robust results. The large non-cancer population, the representation of services from inpatient and community settings (both private and public providers), and the inclusion of data from younger age groups in our study are further strengths. The applied nature of our study design while a strength also resulted in a number of the quality checks being employed only after all of the data had been submitted by the services, and this resulted in the need to further investigate missing values for the insomnia item in the national outcomes program and in future studies.

## Conclusion

PCOC SAS is a valid and reliable patient-reported measure able to be used by patients with advanced disease. The attributes of PCOC SAS allow it to be used as part of routine clinical care, including with younger patients, those with a diagnosis other than cancer, across settings, with individual services (e.g., tertiary providers through to smaller community providers), and as part of a national palliative care outcome programs. Given these attributes, PCOC SAS is distinguished from other scales that aim to measure what affects patients with advanced illness. It is recommended for use in palliative care.

## Supporting information

S1 File (DOCX)

## References

[1] Sleeman KE, de Brito M, Etkind S, Nkhoma K, Guo P, Higginson IJ, et al. The escalating global burden of serious health-related suffering: projections to 2060 by world regions, age groups, and health conditions. The Lancet Global Health. 2019; 7(7):e883–e92. 10.1016/S2214-109X(19)30172-X 31129125 PMC6560023

[2] Pruitt SD, Epping-Jordan JE. Preparing the 21st century global healthcare workforce. BMJ (Clinical research ed). 2005; 330(7492):637–9. 10.1136/bmj.330.7492.637 15774994 PMC554912

[3] Currow DC, Allingham S, Yates P, Johnson C, Clark K, Eagar K. Improving national hospice/palliative care service symptom outcomes systematically through point-of-care data collection, structured feedback and benchmarking. Support. Care in Cancer. 2015; 23(2):307–15. 10.1007/s00520-014-2351-8 25063272 PMC4289012

[4] Kristjanson LJ, Pickstock S, Yuen K, Blight J, Cummins A, Dean AA, et al. Development and Testing of the Revised Symptom Assessment Scale: Final report. Perth, Australia: Edith Cowan University, 1999.

[5] Aoun SM, Monterosso L, Kristjanson LJ, McConigley R. Measuring symptom distress in palliative care: psychometric properties of the Symptom Assessment Scale (SAS). J Palliat Med 2011; 14(3):315–21. 10.1089/jpm.2010.0412 21254812

[6] Terwee CB, Bot SD, de Boer MR, van der Windt DA, Knol DL, Dekker J, et al. Quality criteria were proposed for measurement properties of health status questionnaires. J. Clin. Epidemiol. 2007; 60(1):34–42. 10.1016/j.jclinepi.2006.03.012 17161752

[7] Murtagh FE, Ramsenthaler C, Firth A, Groeneveld EI, Lovell N, Simon ST, et al. A brief, patient- and proxy-reported outcome measure in advanced illness: Validity, reliability and responsiveness of the Integrated Palliative care Outcome Scale (IPOS). Palliat. Med. 2019; 33(8):1045–57. 10.1177/0269216319854264 31185804 PMC6691591

[8] Sleeman KE, Higginson IJ. A psychometric validation of two brief measures to assess palliative need in patients severely affected by multiple sclerosis. JPSM. 2013; 46(3):406–12. 10.1016/j.jpainsymman.2012.08.007 23195389

[9] Clapham S, Allingham S, Daveson B, Eagar K, Blackburn P, Morris D, et al. Measuring patient-reported symptom distress in palliative care. When is a patient self-report used versus proxy report? Results of a national study. Wollongong, Australia: Palliative Care Outcomes Collaboration, Australian Health Services Research Institute (AHSRI), University of Wollongong, 2020.

[10] Clapham S, Holloway A, PCOC. Palliative Care Outcomes Collaboration clinical manual. Wollongong, Australia: Australian Health Services Research Institute (AHSRI), University of Wollongong, 2018.

[11] Masso M, Allingham SF, Banfield M, Johnson CE, Pidgeon T, Yates P, et al. Palliative Care Phase: inter-rater reliability and acceptability in a national study. Palliat. Med. 2015; 29(1):22–30. 10.1177/0269216314551814 25249239

[12] Eagar K, Green J, Gordon R. An Australian casemix classification for palliative care: technical development and results. Palliat. Med. 2004; 18(3):217–26. 10.1191/0269216304pm875oa 15198134

[13] Abernethy AP, Shelby-James T, Fazekas BS, Woods D, Currow DC. The Australia-modified Karnofsky Performance Status (AKPS) scale: a revised scale for contemporary palliative care clinical practice [ISRCTN81117481]. BMC Palliat. Care. 2005; 4:7. 10.1186/1472-684X-4-7 16283937 PMC1308820

[14] Masso M, Allingham SF, Johnson CE, Pidgeon T, Yates P, Currow D, et al. Palliative Care Problem Severity Score: reliability and acceptability in a national study. Palliat. Med. 2016; 30(5):479–85. 10.1177/0269216315613904 26503920

[15] Fayers PM, Machin D. Scores and measurements: validity, reliability, sensitivity. Quality of life: the assessment, analysis and interpretation of patient reported outcomes. 2nd ed. Chichester: John Wiley & Sons; 2007. pp. 77–108.

[16] Landis JR, Koch GG. The measurement of observer agreement for categorical data. Biometrics. 1977; 33(1):159–74. 843571

[17] Kincaid JP, Fishburne RP, Rogers RL, Chissom BS. Derivation of new readability formulas (automated readability index, fog count, and flesch reading ease formula) for Navy enlisted personnel. Research Branch Report 8–75. Millington, TN: Naval Air Station Memphis, Institute for Simulation and Training. 1975.

[18] Streiner DL, Norman GR. Health measurement scales. A practical guide to their development and use. 3rd ed. New York: Oxford University Press; 2003.

[19] Sprangers MA, Schwartz CE. Integrating response shift into health-related quality of life research: a theoretical model. Soc. Sci. Med. 1999; 48(11):1507–15. 10.1016/s0277-9536(99)00045-3 10400253

[20] Schwartz CE, Sprangers MA. Methodological approaches for assessing response shift in longitudinal health-related quality-of-life research. Soc. Sci. Med. 1999; 48(11):1531–48. 10.1016/s0277-9536(99)00047-7 10400255

[21] Hagedoorn M, Sneeuw KC, Aaronson NK. Changes in physical functioning and quality of life in patients with cancer: response shift and relative evaluation of one’s condition. J. Clin. Epidemiol. 2002; 55(2):176–83. 10.1016/s0895-4356(01)00438-3 11809356

