# Supplementary material for: The PCOC Symptom Assessment Scale (SAS): A valid measure for daily use at point of care and in palliative care programs
Source: PLoS One. 2021 Mar 25;16(3):e0247250. doi: 10.1371/journal.pone.0247250 (PMC7993777; doi:10.1371/journal.pone.0247250)
Supplement: S1 File — (DOCX) [file pone.0247250.s001.docx]

| 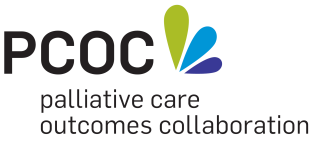 | | | | (Please complete or affix Label here)  UPI:  Surname  First name:  DOB: | | | | | |
| --- | --- | --- | --- | --- | --- | --- | --- | --- | --- |
| **Symptom Assessment Scale**  Please use this form to tell us about the symptoms that bother, worry or distress you. This information will help us to meet your needs.  **Moderate**  **Severe**  **Mild**  **Absent**     \| **0** \| **1** \| **2** \| **3** \| **4** \| **5** \| **6** \| **7** \| **8** \| **9** \| **10** \| \| --- \| --- \| --- \| --- \| --- \| --- \| --- \| --- \| --- \| --- \| --- \|   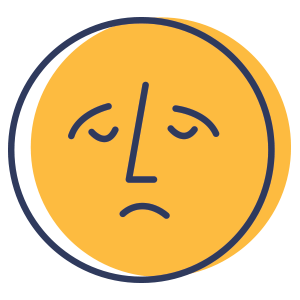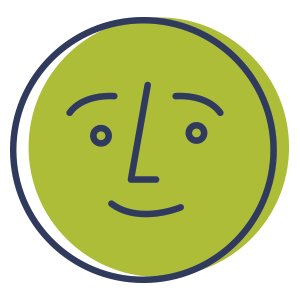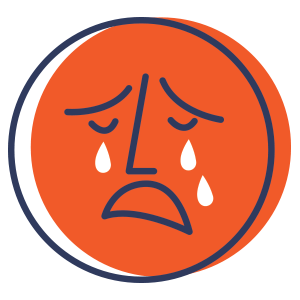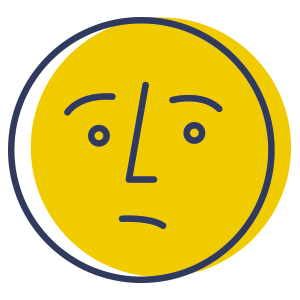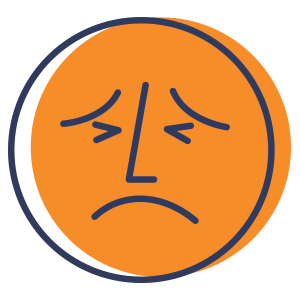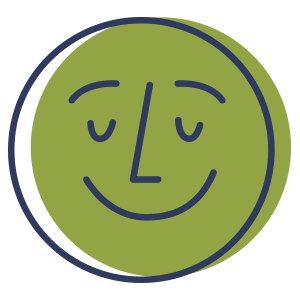   1. Write the day or date in the first row. 2. Use the scale above to choose a number between 0 and 10 that shows how bothered, worried or distressed you are. 3. You can add other symptoms in the blank space at the bottom of the list. | | | | | | | | | |
| **Day or date** |  |  |  |  |  |  |  |  |  |
| Difficulty sleeping |  |  |  |  |  |  |  |  |  |
| Appetite problems |  |  |  |  |  |  |  |  |  |
| Nausea |  |  |  |  |  |  |  |  |  |
| Bowel problems |  |  |  |  |  |  |  |  |  |
| Breathing problems |  |  |  |  |  |  |  |  |  |
| Fatigue |  |  |  |  |  |  |  |  |  |
| Pain |  |  |  |  |  |  |  |  |  |
| Other |  |  |  |  |  |  |  |  |  |
